# Supplementary material for: Understanding the Experience of Service Users in an Integrated Care Programme for Obesity and Mental Health: A Qualitative Investigation of Total Wellbeing Luton
Source: Int J Environ Res Public Health. 2022 Jan 12;19(2):817. doi: 10.3390/ijerph19020817 (PMC8775803; doi:10.3390/ijerph19020817)
Supplement: Supplementary file 1 [file ijerph-19-00817-s001.zip › Supplementary information S1 Interview schedule for service users.pdf]

Title of Project: Evaluating Total Wellbeing Luton: An exploration of service users' experiences.

Interview schedule for service users

Introduction

- Thank participants for agreeing to take part in this interview.
- Provide background to the project: I am interested in your experiences and satisfaction from the service.
- Brief outline of the interview: We will discuss about your experiences from the service and the level of satisfaction. There are no right or wrong answers.
- Explain about the consent procedure, highlight that the interview will be recorded, they do not have to answer any question they do not want to, stress that the participation in voluntary and they can stop the interview any time without giving any reason.
- The findings from the research will be written up for my Ph.D. thesis and may be published in academic / professional journals.
- Confidentiality. Any quotes will be anonymised and I will ensure that there are no issues than can trace the interview with them.
- Before we start, participants will be asked to sign two copies of the consent form. One of them and one for the project's file.
- Any questions or concerns before we start the interview?

Introduction

- Can you tell me a little about yourself?

Understanding service users' contact with the service

- Where did you hear about Total Wellbeing? Where did you get this information from? (GP/ health professionals , leaflets, advertisements)
- What service(s) did you use? Tell me about the support you got from [name of the service]
- What information were you given about Total Wellbeing before using the service(s)?

Experience

- Can you tell me a bit about your experience with the [name of the service]?
- What did you value the most about the support the [name of the service] provided?
- Do you feel that the service is able to address your needs and supports you?
- Do you feel that the support that you receive is tailored based on your needs?
- How involved are/were you in the decision about your support? Are/Were involved

as much as you wanted to be in decisions about your support?

- How would you describe the communication between you and staff?
- What is working well in [name of the service]? In what area is it good? Tell me what is good and why
- What is not working well in [name of the service]? In what areas could it be better? Tell me what is not working well and why
- What would like to receive from the service?
- What would change in the [name of the service]?

Impact on wellbeing

- Have you noticed any changes to your health and wellbeing as a result of seeing the [name of the service] team?
  If Yes, describe how these have benefitted you?
  (change in mood?, changes in physical health?, changes in your social life?)

Referral to other services?

- Did the [name of the service] offer/refer you to any other services of TWL?
  If Yes, what was it?
  Could you please describe your experience? How did it impact you?
