# Supplementary material for: Understanding the Experience of Service Users in an Integrated Care Programme for Obesity and Mental Health: A Qualitative Investigation of Total Wellbeing Luton
Source: Int J Environ Res Public Health. 2022 Jan 12;19(2):817. doi: 10.3390/ijerph19020817 (PMC8775803; doi:10.3390/ijerph19020817)
Supplement: Supplementary file 1 [file ijerph-19-00817-s001.zip › Supplementary information S2 Summary of qualitative themes.pdf]

Summary of qualitative themes

Theme	Subtheme	Supporting Quote
A patient-centered system	Offers holistic support	"They are there for everybody, and a wide range of help and services are available. Particularly now when you know things, over the last year or so with Covid-19, uncertainties and mental health problems, either to make you feel as though you're not alone." (Service user 1)
	Patient empowerment	"They gave us an opportunity to discuss and share ideas, back and forth with each other. And that was quite good really. They didn't dominate the class and gave us an opportunity." (Service user 5)
Supports behaviour change	Knowledge acquisition	"I was really interested in reading food labels, I had health problems, I had to go into FOD map diet. They really help with that. I learned how to read the food labels" (Service user 14)
	Increased motivation to perform new healthy behaviours	"If you're doing it on your own, you have to have the motivation to do it. If you've got a class to go to, then, it's in your schedule, and you will go. […] The physical benefit, but it's also the social benefit as well, because there's a good group of people there. Got to know each other over the years and, yeah, we have a good laugh. […] I think because doing it in a group makes you go there. […]Monday morning, I go to a gym it's a regular habit. " (Service user 13)
	Enabling self-management	"The therapist showed me different techniques, things like a journal. It's called a worries diary, I read it every day, and if I ever have a stressful day I still go back to that. And so I still go back to all those still now." (Service user 3)
Valued social support	Ongoing health professional support	"It's been lockdown and I've kind of gone off track. And, you know, he was always able to see and not just like a couple of minutes and say… "Do this, do this, do this", it wasn't like that, he gave me a lot of time, even on the phone." (Service user 6)
	Camaraderie among other service users	"The camaraderie. When I first came, we barely had coffee once every few weeks. And then we start coming down here to get a coffee, which is lovely. They lift you emotionally." (Service user 12)
	More follow – up is needed	"And then there should be a report of your progress. Is it working or is it not working? To have measurements, is it working or is it not working? […] After

		the program, after six months down the line, they should ask "how are you getting on?" It is a tester. What happened after the tester? And you'll always find some people. If you have 20, you might end up with five, who just want to keep going. Yes, it makes a difference to these five and should be worth it." (Service user 5)
Communication is key	Active listening and responding to needs	"They listened to everything I said and they made me feel very comfortable to open up because it's not something that I've been able to do before." (Service user 2)
	Online vs face-to-face	"I think the online courses, it's all right, and they give you information base. The online course doesn't answer some of your questions. I mean you can talk to someone face-to-face and say, well, I've got this. And that can give you a direct answer rather than you're thinking, well, is that right is that wrong." (Service user 8)
	Unaware of integration	"I didn't know it was a holist program. I think it would be nice that they explained what they were in the beginning. Saying, we could do this. Nobody ever said that to me. We do this, this, this, and this, you gradually find out for yourself really, just asking questions. I wasn't aware of what they did until you graduate. It would be nice if you were sat down and someone spoke to you and told you: "We can help you completely." No one ever said that; that would be nice and useful." (Service user 11)
	Poor visibility of the service	"I would say I'd highly recommend it. But it's a lot of hard work in my heart to get the information. And because I am a person like that. I know how to get information. I am quite proactive in doing things. I could access it, but for a lot of people that would have been very difficult to do. […] I think when you get into it, it works. That's just how you know it's there…. for me the information of what they can do for you is not obvious." (Service user 11)
Flexible referral process	Health professional encouragement	"Because my doctor had concerns over my BMI. So, and he kept on saying to me, like you've got to do something, and then earlier this year I went to see. And he said to me, you've got to accept some help. So I said, All right, fair enough. So that's what I came and I applied for the course, I got recommended for the course." (Service user 8)

	Ability to self-refer	"And then, after all that lockdown. I contacted the gym again. I'm a member there and I said, "Look, I really want to get back into exercise again and I need some help." And so they gave me [name of member of staff], and she was brilliant. We met twice in the gym and we went to a program and then lock-down again. So, it has been wonderful. Really, really good." (Service user 11)
	Ease of access	"They've got some nurses at the surgery that I use, and they put me forward to the nurse who then rang me and said, they've got someone to do with people losing weight and stuff like that. And they then recommended me and someone contacted me about it." (Service user 8)
Positive impact on life	Improved health and well-being	"I have. I managed to lose two stone, my blood pressure in normal now, the nurse said she was really shocked, that is beautiful, whatever you're doing, keep it up. So, she was really good, I saw a lot of changes within myself." (Service user 6)
	Changes in social life	"I definitely go out and socialize more, and how I was feeling before, I think before I went out and it was make not to want to go. But now, I like what I've learned in my mind and make sure that I don't avoid situations just because of how I'm feeling because I'm never going to overcome it if I keep avoiding them. Definitely, like, I'm more willing to go and socialize." (Service user 2)
	Optimistic outlook on life	"They helped me physically, mentally and emotionally. So I will say they changed my life. I was suffering from a lot of health problems and rapidly a lot of it improved." (Service user 6)
